# Identification of Metabolites of the Cardioprotective Alkaloid Dehydrocorydaline in Rat Plasma and Bile by Liquid Chromatography Coupled with Triple Quadrupole Linear Ion Trap Mass Spectrometry

**DOI:** 10.3390/molecules22101686

**Published:** 2017-10-10

**Authors:** Huanyu Guan, Kaitong Li, Xiaoming Wang, Xiaomei Luo, Meifeng Su, Wenting Tan, Xiaoyan Chang, Yue Shi

**Affiliations:** 1 Institute of Medicinal Plant Development, Chinese Academy of Medical Sciences and Peking Union Medical College, Beijing 100193, China; guanhuanyu630@163.com (H.G.); likaitong@joinn-lab.com (K.L.); lmlwxm123@163.com (X.W.); Luoxiaomei1019@163.com (X.L.); 20160931861@bucm.edu.cn (M.S.); 18811790612@163.com (W.T.); changxiaoyan1234@163.com (X.C.); 2 School of Pharmaceutical Sciences, Guizhou Medical University, Guiyang 550004, China

**Keywords:** LC–MS/MS, dehydrocorydaline, *Corydalis yanhusuo*, metabolites, cardioprotection

## Abstract

Dehydrocorydaline (DHC), a quaternary alkaloid from *Corydalis yanhusuo*, has been demonstrated to be the active constituent in the treatment of coronary heart disease. In this study, a high-performance liquid chromatography–electrospray ionization–triple quadrupole linear ion trap mass spectrometry (HPLC–ESI–QTRAP MS) technique was used to identify DHC metabolites in plasma and bile after oral administration of DHC to rats. A total of 18 metabolites (M1 to M18) were identified and characterized by LC–MS/MS in the positive ion mode. These 18 metabolites were all present in rat bile, while only 9 were detected in plasma. *O*-demethylation, hydroxylation, di-hydroxylation, glucuronidation of *O*-demethyl DHC, sulfation of *O*-demethyl DHC and di-hydroxylation of dehydro-DHC were the major metabolic pathways of DHC. This is the first time that these metabolites of DHC have been identified in rat plasma and bile, which provides useful information for further analysis of the biotransformation of DHC and other quaternary protoberberine-type alkaloids.

## 1. Introduction

*Corydalis yanhusuo* W.T. Wang, a perennial herb of the Papaveraceae family, is well known as a traditional Chinese medicine (TCM) [1]. The preparation of *C. yanhusuo* extract, as Ke-Da-Ling tablets, has been used to treat cardiovascular disease in modern clinical practice [2]. A quaternary protoberberine alkaloid of *C. yanhusuo*, namely, dehydrocorydaline (DHC), has been demonstrated to be the active constituent in the treatment of coronary heart disease [3]. Structurally, DHC is comprised of 5,6-dihydrodibenzo [*a*,*g*] quinolizinium (C_17_H_14_N^+^) [4], a methyl group at C-13 and four methoxyl groups at C-2, -3, -9 and -10. Pharmacological studies have revealed that DHC can improve hypoxia tolerance in rats, expand the coronary artery and increase the blood flow in this artery [5]. Through the previously established fingerprint-efficacy relationship between chemical fingerprints of quaternary ammonium alkaloids from *C. yanhusuo* and its cardioprotection efficiency, we found that DHC is one of the main effective components of this TCM and can be adopted as a reference component for quality control of *C. yanhusuo* [6]. In addition, DHC is also used as a reference index to evaluate the quality of Ke-Da-Ling tablets [7].

The in vivo metabolism of this drug plays an important role in the production of active metabolites and the generation of the resulting activities. Radioactive tracing experiments showed that DHC was subjected to the first-pass effect in the liver and was distributed mainly in the digestive tract, liver and kidney after oral administration of ^14^C-dehydrocorydaline to rats. The compound was metabolized by *O*-demethylation and then by glucuronidation [8]. However, the metabolic fate of DHC was not completely established.

In our previous study, the in vivo metabolism of *C. yanhusuo* extract in rat plasma was analyzed by high-performance liquid chromatography coupled to mass spectrometry (HPLC–MS), which revealed that 10 bioactive components of *C. yanhusuo* were present in the blood [9]. Additionally, we conducted a comparative pharmacokinetic analysis of DHC after administration of *C. yanhusuo* and pure DHC using LC–MS/MS [10], and found that the oral administration of *C. yanhusuo* extract accelerated the absorption of DHC and slowed down its elimination.

With the development of liquid chromatography coupled with mass spectrometry (LC–MS) and data acquisition software, the identification of metabolites present at low concentrations in complex biological matrices has become easier to achieve. To further evaluate the in vivo metabolism of DHC and to obtain more information for pharmacological research, a high-performance liquid chromatography–electrospray ionization–triple quadrupole linear ion trap mass spectrometry (HPLC-ESI-QTRAP MS) method was developed to analyze the metabolites of DHC in rat plasma and bile. A total of 18 metabolites were identified using the multiple reaction monitoring–information-dependent acquisition–triggering enhanced product ion (MRM-IDA-EPI) mode.

## 2. Results and Discussion

### 2.1. Optimization of HPLC Conditions

To improve the resolution and intensities of chromatographic peaks, various compositions of the mobile phase (methanol, acetonitrile, ammonium acetate and formic acid) were tested to optimize the chromatographic conditions. As a result, acetonitrile and water (containing 0.8% formic acid and 10 mM ammonium acetate; 25:75 *v*/*v*) were employed as the mobile phase because of the best separation and an abundant signal.

### 2.2. Optimization of Sample Preparation

Liquid–liquid extraction (ethyl acetate and water-saturated *n*-butanol), protein precipitation (acetone, acetonitrile and methanol) and solid-phase extraction (SPE) were evaluated to obtain the metabolites. Compared with liquid–liquid extraction, SPE could ensure the simultaneous extraction of target compounds. The higher intensities of target peaks were obtained via SPE compared to using protein precipitation. Moreover, methanol containing 2% formic acid was selected as the elution solvent for SPE. To determine the optimum volume of methanol containing 2% formic acid to elute target compounds from the SPE cartridge, the elution was carried out two times with 1.0 mL of methanol containing 2% formic acid for each time. It was concluded that 1.0 mL of methanol containing 2% formic acid was sufficient to wash out the target compounds.

### 2.3. Identification of DHC

**Figure 1:**
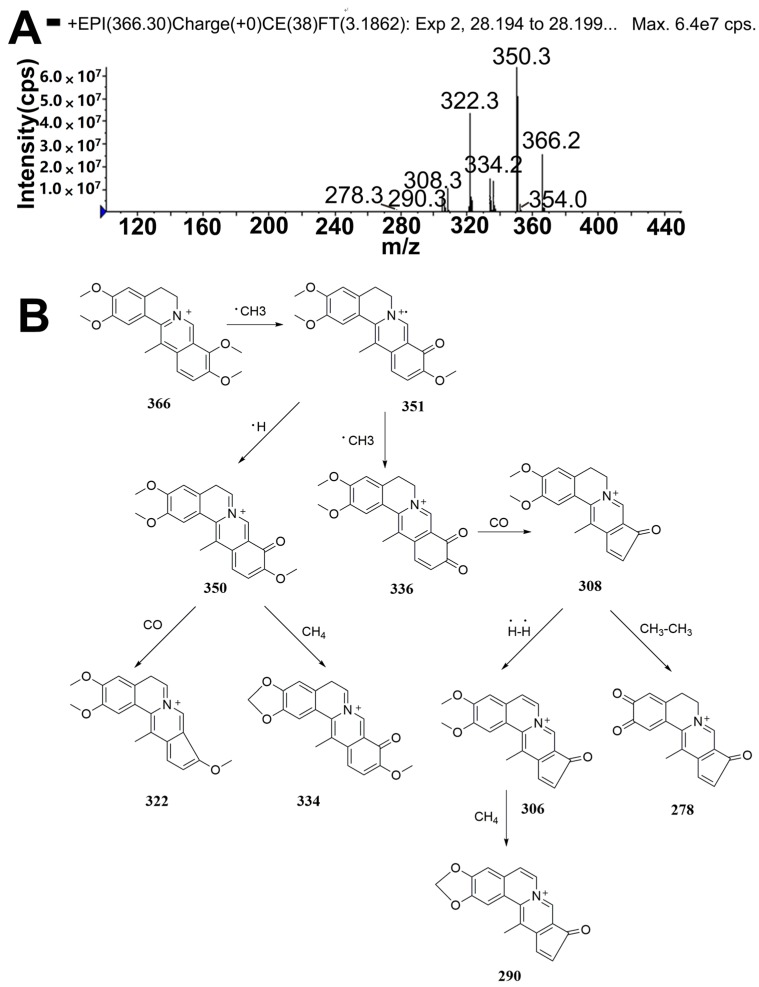
Mass spectrometry (MS^2^) spectrum (**A**) of dehydrocorydaline (DHC) and its proposed fragmentation pathways (**B**).

The molecular ion [M]^+^ of DHC in the positive mode was observed at *m*/*z* 366, corresponding to the cationic form of DHC. The fragment ions at *m*/*z* 351, 350, 334, 322 and 308 were assigned as [M − CH_3_]^+^, [M − CH_3_ − H]^+^, [M − CH_3_ − H − CH_4_]^+^, [M − CH_3_ − H − CO]^+^ and [M − CH_3_ − CH_3_ − CO]^+^, respectively. Moreover, *m*/*z* at 290 was fragmented by dehydrogenation [11] and loss of CH_4_ [12] from the fragment ion with *m*/*z* 308. The fragment ion with *m*/*z* 278 was also generated by the loss of CH_3_CH_3_ from the ion *m*/*z* 308. The fragmentation scheme for DHC is proposed in Figure 1.

### 2.4. Identification of Metabolites

**Figure 2:**
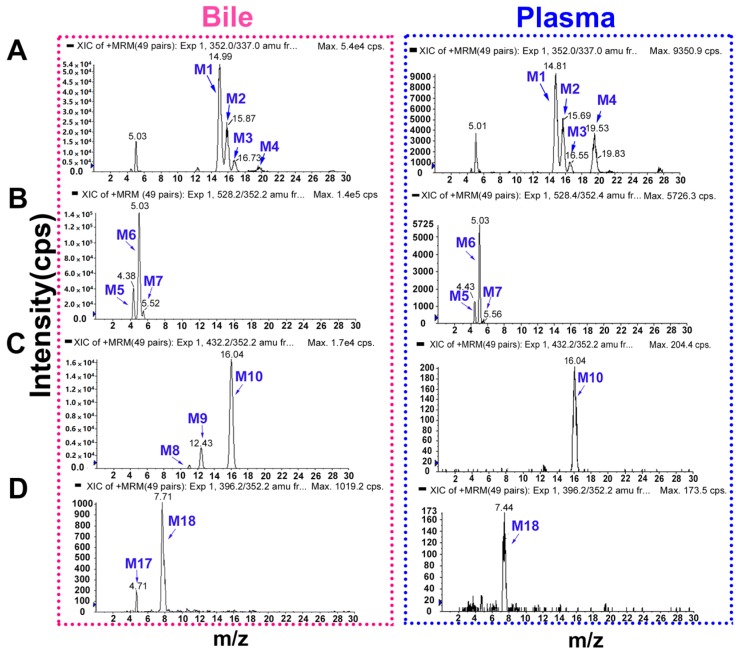
The extracted ion chromatograms (XICs) of the dehydrocorydaline (DHC) metabolites in bile and plasma. (**A**) *O*-demethylated metabolites (M1–M4); (**B**) *O*-demethylated metabolites followed by glucuronidation (M5–M7); (**C**) *O*-demethylated metabolites followed by sulfation (M8–M10); and (**D**) metabolites due to di-hydroxylation and dehydrogenation (M17 and M18).

**Figure 3:**
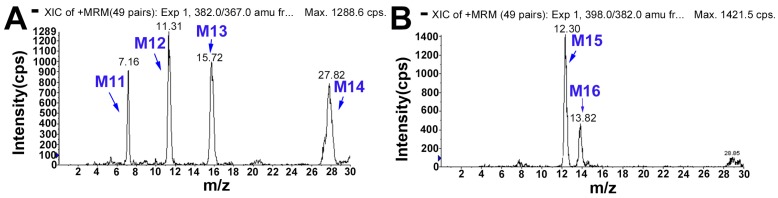
The extracted ion chromatograms (XICs) of dehydrocorydaline (DHC) metabolites in bile. (**A**) Hydroxylated metabolites, and (**B**) di-hydroxylated metabolites.

On the basis of the generic biotransformation table built in Metabolite ID (MetID), 49 MRM transitions were monitored during the complete analytical run. Once a listed metabolite was found by MRM, the EPI spectral acquisition of the metabolite was triggered. A total of 18 metabolites detected in rat bile and plasma are listed in Table 1. The MRM extracted ion chromatograms (XICs) of the metabolites of DHC in bile and plasma are shown in Figure 2 and Figure 3. Compared with the peaks in the corresponding blank sample, 18 additional peaks in rat bile and 9 in plasma were observed and presumed to be metabolites. The observed metabolites were generated by *O*-demethylation, *O*-demethylation followed by glucuronidation, *O*-demethylation followed by sulfation, di-hydroxylation and dehydrogenation, di-hydroxylation and hydroxylation.

### 2.5. Structure Elucidation of DHC Metabolites

#### 2.5.1. Metabolites Produced by *O*-demethylation (M1, M2, M3 and M4)

**Figure 4:**
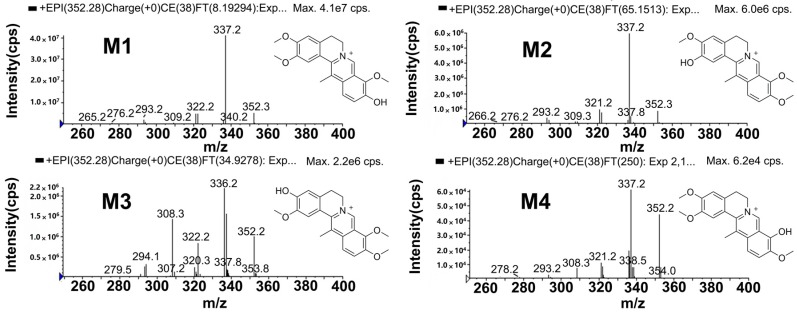
The mass spectrometry (MS^2^) spectra of 13-methyl-dehydrocorydalmine (**M1**), 13-methyl-columbamine (**M2**), dehydrocorybulbine (**M3**) and 13-methyl-palmatrubine (**M4**).

M1, M2, M3 and M4 were detected at approximately 15.0, 15.9, 16.7 and 19.5 min, respectively. Each of these exhibited a molecular ion at *m*/*z* 352, which indicated the loss of one methyl group (14 Da) from DHC. The product ions (Figure 4 and supplementary material Figure S1) at *m*/*z* 337 and 336 were generated by the loss of CH_3_ (−15 Da) and CH_4_ (−16 Da) from the molecular ion. The product ions at *m*/*z* 322 and 309 were formed by the loss of CH_3_ (−15 Da) and CO (−28 Da) from the product ion at *m*/*z* 337. The product ions at *m*/*z* 309 further lost a molecule of CH_4_ to generate the product ion at *m*/*z* 293. Similarly, the product ion at *m*/*z* 336 lost a CO fragment to yield the product ion at *m*/*z* 308. To further identify the chemical position that was *O*-demethylated, the XICs of 13-methyl-dehydrocorydalmine, dehydrocorybulbine, 13-methyl-palmatrubine and palmatine were compared to those of M1–M4. The retention time (*t*_R_) of 13-methyl-dehydrocorydalmine, dehydrocorybulbine and 13-methyl-palmatrubine were 15.1, 16.9 and 19.8 min (Figure S1), respectively, which matched those of M1, M3 and M4. DHC was not demethylated at C-13 in rat bile and plasma, as indicated by the absence of the signal peak of palmatine (*t*_R_ = 21.5 min) shown in Figure S1. The 2-*O*-methyl group of DHC was more unstable in the metabolic pathway of *O*-demethylation. M2 was presumed to be generated by *O*-demethylation of DHC at C-2. Accordingly, M1, M2, M3 and M4 were identified as 13-methyl-dehydrocorydalmine, 13-methyl-columbamine, dehydrocorybulbine and 13-methyl-palmatrubine.

#### 2.5.2. Metabolites Produced by Glucuronidation of *O*-Demethyl-DHC (M5, M6 and M7)

**Figure 5:**
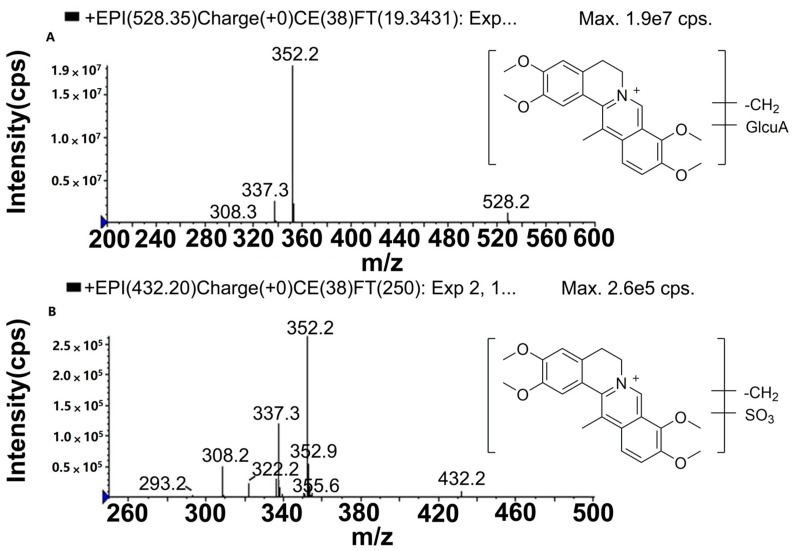
The mass spectrometry (MS^2^) spectra of *O*-demethylated metabolites followed by glucuronidation (**A**: M5, M6 and M7) and sulfation (**B**: M8, M9 and M10).

M5 (*t*_R_ = 4.4 min), M6 (*t*_R_ = 5.0 min) and M7 (*t*_R_ = 5.5 min) had molecular ions (Figure 5) at *m*/*z* 528, which were 176 Da heavier than M1, M2, M3 or M4. The product ion at *m*/*z* 352, generated by the loss of C_6_H_8_O_6_ from the molecular ion, implied that glucuronidation occurred for M1, M2, M3 or M4. The fragmentation behaviors of M5, M6 and M7 were similar to those of the glucuronide conjugates of *O*-demethyl-palmatine [11]. Therefore, M5, M6 and M7 were deduced to be the glucuronide products of *O*-demethyl-DHC.

#### 2.5.3. Metabolites Produced by Sulfation of *O*-demethyl-DHC (M8, M9 and M10)

M8 and M9 detected in bile and M10 detected in both bile and plasma were eluted at *t*_R_ values of 10.9, 12.4 and 16.0 min, respectively. Each of these exhibited a molecular ion at *m*/*z* 432 (Figure 5), which was increased by 80 Da compared with M1, M2 or M3. The molecular ion at *m*/*z* 432 produced five major product ions at *m*/*z* 352, 337, 322, 308 and 293. The product ion at *m*/*z* 352 was formed by the loss of SO_3_ from the molecular ion, and further lost a CH_3_ group to generate the product ion at *m*/*z* 337. The product ion at *m*/*z* 322 originated from the loss of a CH_3_ group by the product ion at *m*/*z* 337. The product ion at *m*/*z* 308 was ascribed to [M − SO_3_ − CH_3_ − H − CO]^+^, from which the ion with *m*/*z* 293 was formed through the loss of a CH_3_ group. The five product ions mentioned above were present in the MS/MS spectrum of M1, M2, M3 and M4. The molecular and fragment ions of M8, M9 and M10 were 14 Da higher than the corresponding ions of sulfated conjugates of *O*-demethyl-palmatine [13]. Accordingly, M8, M9 and M10 were assigned as the sulfated conjugates of *O*-demethyl-DHC.

#### 2.5.4. Metabolites Produced by Hydroxylation (M11, M12, M13 and M14)

**Figure 6:**
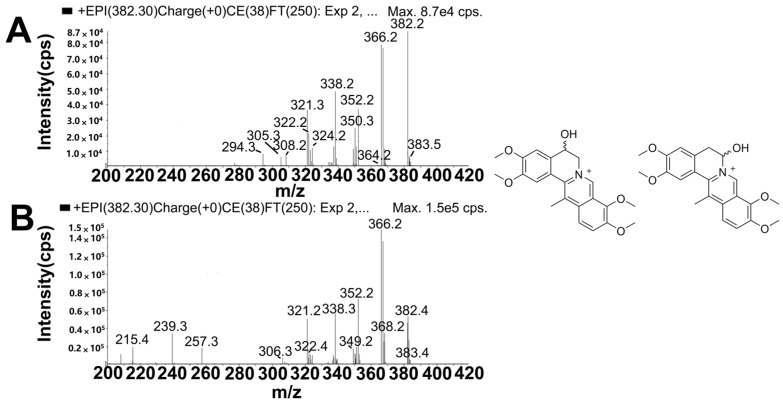
The mass spectrometry (MS^2^) spectra of hydroxylated metabolites (**A**: M11; **B**: M12, M13 and M14).

M11, M12, M13 and M14 in rat bile were eluted at retention times of 7.2, 11.3, 15.7 and 27.8 min, and all displayed the molecular ion at *m*/*z* 382, which was 16 Da higher than that of DHC and 14 Da higher than that of mono-hydroxylated metabolites of palmatine [14]. The product ions (Figure 6 and Supplementary Figure S2), specifically at *m*/*z* 367 ([M − CH_3_]^+^), 366 ([M − CH_3_ − H]^+^), 350 ([M − CH_3_ − H − CH_4_]^+^), 338 ([M − CH_3_ − H − CO]^+^) and 324 ([M − CH_3_ − CH_3_ − CO]^+^), were also 16 Da higher than the corresponding fragment ions of DHC. These results indicated that M11, M12, M13 and M14 may have been the mono-hydroxylated products of DHC. In addition, the product ion of M11 at *m*/*z* 364 ([M − H_2_O]^+^) and those of M12, M13 and M14 at *m*/*z* 349 ([M − CH_3_ − H_2_O]^+^), 321 ([M − CH_3_ − H_2_O − CO]^+^) and 306 ([M − CH_3_ − H_2_O − CO − CH_3_]^+^) indicated that [M]^+^ underwent dehydration between C-5 and -6. Therefore, M11, M12, M13 and M14 were identified as 5- or 6-hydroxyl-DHC and its stereoisomer.

#### 2.5.5. Metabolites Produced by Di-Hydroxylation (M15 and M16)

**Figure 7:**
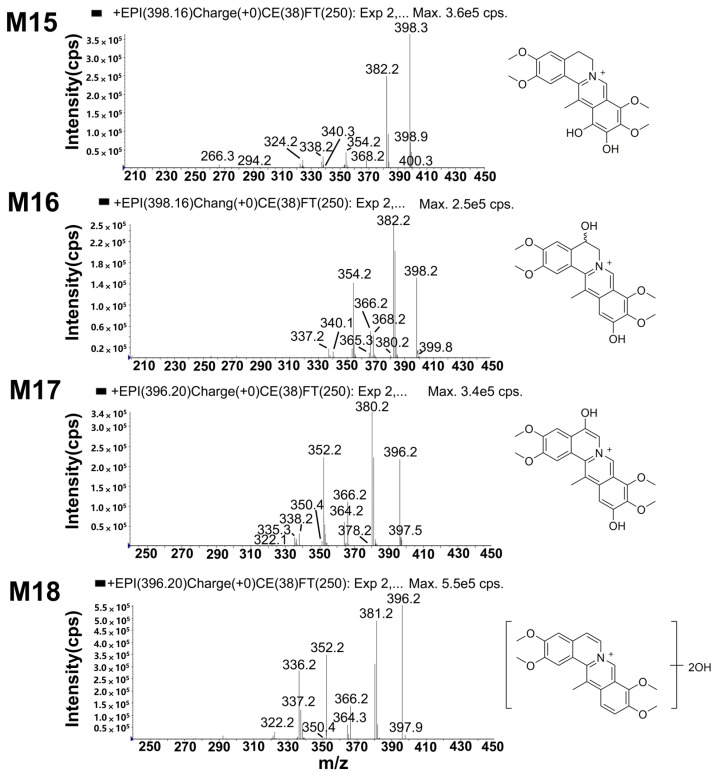
The mass spectrometry (MS^2^) spectra of di-hydroxylated metabolites (M15 and M16) and metabolites (M17 and M18) produced by di-hydroxylation and dehydrogenation.

**Figure 8:**
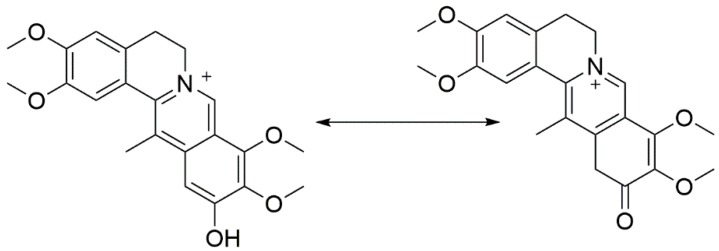
The arrangement of 11-hydroxyl dehydrocorydaline (DHC).

Both M15 (*t*_R_ = 12.3 min) and M16 (*t*_R_ = 13.8 min) displayed a molecular ion at *m*/*z* 398, which was 32 Da heavier than that of DHC and 14 Da heavier than that of di-hydroxylated metabolites of palmatine [15]. The fragment ions (Figure 7) at *m*/*z* 383 ([M − CH_3_]^+^), 382 ([M − CH_4_]^+^), 354 ([M − CH_3_ − H − CO]^+^) and 340 ([M − CH_3_ − CH_3_ − CO]^+^ were increased by 32 Da compared with those of DHC. Thus, M15 and M16 were identified as the di-hydroxylates of DHC. Moreover, the fragment ions of M16 (Figure S3) at *m*/*z* 380, 365 and 337 were assigned as [M − H_2_O]^+^, [M − H_2_O − CH_3_]^+^ and [M − H_2_O − CH_3_ − CO]^+^, which indicated that M16 underwent dehydration to generate a double bond between C-5 and -6. These results implied that one of the hydroxylation positions of M16 was either C-5 or -6. According to a previous report [14], the position order of a hydroxylation reaction is C-5, -11 and -6; the hydroxylation of C-5 is easier than that of the other positions. M16 was tentatively identified as 5,11-di-hydroxyl-DHC. The fragment ions of M15 at *m*/*z* 338 ([M − 2CH_3_ − CO − 2H]^+^), 322 ([M − 2CH_3_ − CO − 2H − CH_4_]^+^), 294 ([M − 2CH_3_ − CO − CH_4_ − 2H − CO]^+^) and 266 ([M − 2CH_3_ − CO − CH_4_ − 2H − 2CO]^+^) showed the loss of two hydrogen atoms, a characteristic feature of the collision spectrum of M15, which indicated that the two hydroxyl modifications occurred at the D-ring. The proposed fragmentation pathway is shown in Figure S4. The first hydroxyl group at C-11 underwent arrangement to saturate C-12 adjacent to the aromatic (sp^2^) carbon atom, which made C-12 easier to be oxidized (Figure 8).

#### 2.5.6. Metabolites Produced by Di-Hydroxylation and Dehydrogenation (M17 and M18)

M17 in bile and M18 in both bile and plasma were eluted at approximately 4.7 and 7.7 min, respectively. Each of these exhibited a molecular ion at *m*/*z* 396 (Figure 7), which was 2 Da lighter than M15 or M16. The results indicated that dehydrogenation had occurred to M17 and M18. M17 and M18 were deduced to be dehydrogenated products of di-hydroxyl-DHC. The fragment ions of M17 at *m*/*z* 380, 366, 364, 352 and 338 were 2 Da less than the corresponding fragment ions of M16. Moreover, the fragment ions of M17 at *m*/*z* 378 ([M − CH_4_ − H_2_]^+^), 350 ([M − CH_4_ − H_2_ − CO]^+^) and 335 ([M − CH_4_ − H_2_ − CO − CH_3_]^+^) indicated that oxidation occurred at C-5 and -11. Accordingly, M17 was a dehydrogenation product of M16, namely, 5,11-di-hydroxylated-5,6-dehydro-DHC. Its proposed fragmentation pathway is shown in Figure S5. 

### 2.6. A Summary of Metabolic Pathways for DHC

**Figure 9:**
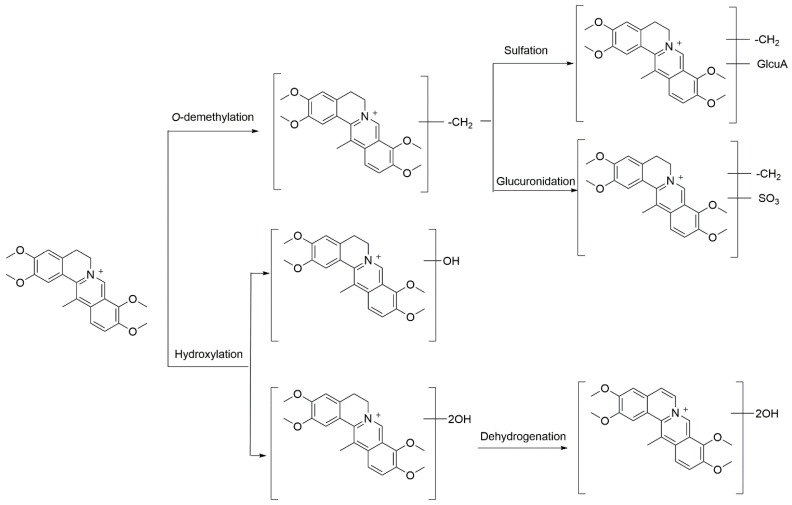
Proposed metabolic pathways of dehydrocorydaline (DHC) in rats.

According to the above analysis results, DHC undergoes *O*-demethylation (M1, M2, M3 and M4), *O*-demethylation followed by glucuronidation (M5, M6 and M7), *O*-demethylation followed by sulfation (M8, M9 and M10), hydroxylation (M11, M12, M13 and M14), di-hydroxylation (M15 and M16), and di-hydroxylation and dehydrogenation (M17 and M18). In total, 18 metabolites were detected in the bile and 9 were identified in plasma. The proposed metabolic pathways of DHC is shown in Figure 9.

## 3. Experimental Section

### 3.1. Materials and Reagents

The standard references DHC, 13-methyl-dehydrocorydalmine, dehydrocorybulbine, 13-methyl-palmatrubine and palmatine were isolated and purified from the dried tuber of *C. yanhusuo* in the authors’ laboratory. The purity of the isolated compounds was found to be above 98.0% as determined by HPLC with a photodiode array detector. HPLC-grade acetonitrile and methanol were purchased from Honeywell Burdick & Jackson Company (Mexico City, Mexico). Formic acid (LC–MS grade) was purchased from Fisher Scientific (Thermo Fisher, Geel, Belgium). Ammonium acetate (HPLC-grade) was purchased from ROE Scientific Inc. (Newark, NJ, USA). Water was deionized using a Milli-Q water purification system (Millipore, Milford, MA, USA). All other reagents were of analytical grade.

### 3.2. Instruments and LC–MS/MS Conditions

An Agilent 1100 system (Agilent Technologies, Santa Clare, CA, USA), consisting of a vacuum degasser, a quaternary pump, a column oven and an auto-sampler, was used coupled to an API 3200 QTRAP mass spectrometer (Applied Biosystems/MDS SCIEX, Concord, ON, Canada). The MetID database (version 1.4.1) was from Applied Biosystems.

Chromatographic separation was achieved on a Diamonsil C18 analytical column (4.6 mm × 250 mm, 5 μm; Dikma Corporation, Beijing, China) eluted with a mobile phase of acetonitrile and water (containing 0.8% formic acid and 10 mM ammonium acetate; 25:75 *v*/*v*) at a flow rate of 1 mL/min. The column temperature was set at 25 °C and the injection volume was 30 μL.

The MS parameters were as follows: ESI was performed in the positive ionization mode; curtain gas (CUR): 10.0 psi; collision gas (CAD): medium; ion spray (IS) voltage: 5500 V; source temperature: 450 °C; GS1: 70 psi and GS2: 60 psi; declustering potential (DP): 70 V; collision energy (CE): 32 eV; entrance potential (EP): 10 V; collision cell exit potential (CXP): 3 V. When MRM–IDA–EPI was performed, the IDA threshold was set at 200 counts per second and the CE in EPI mode was set at 38 eV (CE spread of 15 eV). The EPI scan was operated at a scan rate of 4000 amu/s with dynamic fill in the linear ion trap.

### 3.3. Animal Handling

Male Sprague–Dawley (SD) rats (140–160 g) were obtained from the Department of Laboratory Animal Resources at the National Institutes for Food and Drug Control (certification number: SCXK2014-0013; Beijing, China). The rats were kept in a controlled environment at a temperature of 20–25 °C and a relative humidity of 40–60% on a standard 12/12 h light/dark cycle with free access to water and a normal diet for 7 days. All the experiments were performed according to the National Institutes of Health Guidelines for Animal Research and were approved by the Ethics Committee of the Institute of Medicinal Plant Development, CAMS & PUMC.

For the bile samples, six rats were fasted overnight with free access to water before the test. The rats were anesthetized with 10% (*w*/*v*) chloral hydrate solution (at a dose of 3.0 mL/kg) by intraperitoneal injection and were fixed on a thermo-controlled surgery platform. The bile duct of the rats was cannulated with a polyethylene tube (PE-10) to collect bile samples continuously, as previously described [16]. The blank bile samples were collected within 2 h of bile duct cannulation. The bile samples were collected within 12 h of oral administration of DHC at a dose of 97.5 mg/kg. All the bile samples were stored at −80 °C until analysis.

For the plasma sample, nine rats were fasted overnight, but allowed water ad libitum. Six of the rats were orally administered DHC (97.5 mg/kg, 10 mL/kg). The remaining three rats received the corresponding volume of blank vehicle to obtain the blank plasma. The blood samples (about 0.3 mL) from the retro-orbital plexus were collected into heparinized tubes at 0.25, 0.5, 0.75, 1, 2, 4, 6 and 8 h after dosing and were then centrifuged at 3000 rpm for 10 min. The obtained plasma samples were transferred to an Eppendorf tube and stored at −80 °C for subsequent analysis.

### 3.4. Sample Preparation

Bile samples were thawed at room temperature and centrifuged at 9000 rpm for 10 min. The supernatant was loaded onto a pre-conditioned OASIS HLB column (30 mg, 30 μm, 1 mL; Waters, Milford, MA, USA). Then the SPE column was eluted with 1 mL of water and 1 mL of methanol (containing 2% formic acid, *v*/*v*) in succession. The methanol fraction was collected and evaporated under a gentle flow of nitrogen at 40 °C. The residue was reconstituted in 150 μL of 10% acetonitrile and centrifuged at 12,000 rpm for 10 min. A 30 μL volume of the supernatant was injected into the HPLC–MS/MS system for analysis.

The plasma samples were pooled together to generate a single sample of each matrix. Then, the mixed plasma samples were processed in a similar manner as the bile samples.

## 4. Conclusions

In this study, a LC–MS/MS method was established to detect the metabolites of DHC in plasma and bile samples of rats after oral administration of DHC, an active alkaloid from *C. yanhusuo* used in the treatment of cardiovascular disease. A total of 18 metabolites were identified using the MRM–IDA–EPI mode, and their structures were elucidated on the basis of the retention times of reference standards and characteristic fragment ions. The main metabolic pathways were *O*-demethylation, *O*-demethylation followed by glucuronidation, *O*-demethylation followed by sulfation, di-hydroxylation and dehydrogenation, di-hydroxylation and hydroxylation. The experimental results are useful for further investigation of the biotransformation of DHC in other biological matrices and the metabolism of other quaternary protoberberine-type alkaloids. Moreover, our findings provide valuable information for pharmacological research of DHC.

## Figures and Tables

**Table 1:** Mass spectrometry (MS^2^) data of dehydrocorydaline (DHC) and its metabolites.

No.	Parent Ion *m*/*z*	Retention Time (min)	Formula	Product Ions (MS^2^)
DHC	366	28.4	C_22_H_24_NO_4_^+^	351, 350, 334, 322, 308, 290, 278
M1, M2, M3, M4	352	15.0, 15.9, 16.7, 19.5	C_21_H_22_NO_4_^+^	337, 336, 322, 309, 308, 293
M5, M6, M7	528	4.4, 5.0, 5.5	C_27_H_30_NO_10_^+^	352, 337, 322 308, 293
M8, M9, M10	432	10.9, 12.4, 16.0	C_21_H_22_NSO_7_^+^	352, 337, 322, 308, 293
M11	382	7.2	C_22_H_24_NO_5_^+^	367, 366, 364, 352, 350, 338, 324, 321, 308, 305, 294
M12, M13, M14	382	11.3, 15.7, 27.8	C_22_H_24_NO_5_^+^	367, 366, 352, 350, 349, 338, 324, 321, 306
M15	398	12.3	C_22_H_24_NO_6_^+^	383, 382, 368, 354, 340, 338, 324, 322, 294, 266
M16	398	13.8	C_22_H_24_NO_6_^+^	383, 382, 380, 368, 366, 365, 354, 340, 337
M17	396	4.7	C_22_H_22_NO_6_^+^	381, 380, 378, 366, 364, 352, 350, 338, 335, 322
M18	396	7.7	C_22_H_22_NO_6_^+^	381, 380, 366, 364, 352, 350, 337, 336, 332

## Supplementary Materials

Supplementary materials are available online. Figure S1: The XICs of reference standards (A): (a) 13-methyl-dehydrocorydalmine, (b) dehydrocorybulbine, (c) 13-methyl-palmatrubine and (d) palmatine. Proposed fragmentation pathways (B) for M1, M2, M3 and M4. Figure S2: Proposed fragmentation pathways for M11, M12, M13 and M14. Figure S3: Proposed fragmentation pathways for M16. Figure S4: Proposed fragmentation pathways for M15. Figure S5: Proposed fragmentation pathways for M17.
