# Supplementary figures and images for: Identification of Metabolites of the Cardioprotective Alkaloid Dehydrocorydaline in Rat Plasma and Bile by Liquid Chromatography Coupled with Triple Quadrupole Linear Ion Trap Mass Spectrometry
Source: Molecules. 2017 Oct 10;22(10):1686. doi: 10.3390/molecules22101686 (PMC6151771; doi:10.3390/molecules22101686)

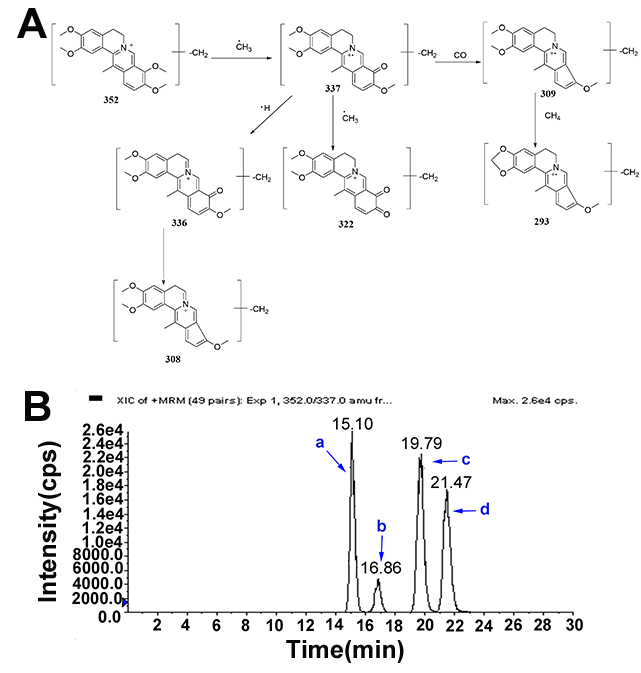

Supplement: Supplementary file 1 [file molecules-22-01686-s001.zip › supporting information/Fig.S1.tif]
